# Combinatorial development of antibacterial Zr-Cu-Al-Ag thin film metallic glasses

**DOI:** 10.1038/srep26950

**Published:** 2016-05-27

**Authors:** Yanhui Liu, Jagannath Padmanabhan, Bettina Cheung, Jingbei Liu, Zheng Chen, B. Ellen Scanley, Donna Wesolowski, Mariyah Pressley, Christine C. Broadbridge, Sidney Altman, Udo D. Schwarz, Themis R. Kyriakides, Jan Schroers

**Affiliations:** 1 Center for Research on Interface Structures and Phenomena, Yale University, New Haven CT 06511, USA; 2 Department of Mechanical Engineering and Materials Science, Yale University, New Haven CT 06511, USA; 3 Department of Biomedical Engineering, Yale University, New Haven CT 06511, USA; 4 Department of Physics, Southern Connecticut State University, New Haven, Connecticut 06515, USA; 5 Department of Molecular, Cellular and Developmental Biology, Yale University, New Haven CT 06520, USA; 6 Department of Chemical and Environmental Engineering, Yale University, New Haven CT 06520, USA; 7 Department of Pathology, Yale University, New Haven CT 06520, USA

## Abstract

Metallic alloys are normally composed of multiple constituent elements in order to achieve integration of a plurality of properties required in technological applications. However, conventional alloy development paradigm, by sequential trial-and-error approach, requires completely unrelated strategies to optimize compositions out of a vast phase space, making alloy development time consuming and labor intensive. Here, we challenge the conventional paradigm by proposing a combinatorial strategy that enables parallel screening of a multitude of alloys. Utilizing a typical metallic glass forming alloy system Zr-Cu-Al-Ag as an example, we demonstrate how glass formation and antibacterial activity, two unrelated properties, can be simultaneously characterized and the optimal composition can be efficiently identified. We found that in the Zr-Cu-Al-Ag alloy system fully glassy phase can be obtained in a wide compositional range by co-sputtering, and antibacterial activity is strongly dependent on alloy compositions. Our results indicate that antibacterial activity is sensitive to Cu and Ag while essentially remains unchanged within a wide range of Zr and Al. The proposed strategy not only facilitates development of high-performing alloys, but also provides a tool to unveil the composition dependence of properties in a highly parallel fashion, which helps the development of new materials by design.

Metallic alloys constitute an essential class of materials having played a vital role since ancient times. In modern days, alloys require a plurality of properties to meet the practical requirements of technological use^1^,^2^. For example, a biomaterial may require strength, processability, and biocompatibility, etc. Metallic alloys used in engineering are most commonly composed of more than three elements to obtain the required properties. To achieve such attributes, completely unrelated development strategies are employed to optimize the composition of a technologically useful alloy. Therefore, it often takes years to identify an alloy composition that integrates multiple properties. Despite the significant improvement in computation capability, current simulation and modeling are typically designed for optimization of individual properties. It remains challenging to predict the composition optimized for unrelated properties. Alloy development has heavily relied on sequential trial-and-error methods of screening, which are time consuming and labor intensive^3^. In contrast to conventional methods, combinatorial approach combining rapid materials fabrication and high-throughput characterization can dramatically increase the pace of materials discovery and optimization. With combinatorial approach, a wide variety of properties can be quick evaluated in different materials classes^4^,^5^,^6^.

Metallic glasses, which are metallic alloys vitrified by rapid cooling, are typical multi-component alloys^7^,^8^,^9^,^10^. They exhibit many remarkable properties such as high strength in combination with plastics-like processability, which are absent in the majority of crystalline metals^10^,^11^,^12^. However, applications of metallic glasses have been limited because the vast majority lacks the required integration of properties to meet technological demands^13^. Recent investigation on some metallic glasses indicates that they can exhibit better antibacterial property than stainless steels, a metal that is commonly used in food production and storage^14^,^15^,^16^. Therefore, it is promising to explore applications for metallic glasses in healthcare ranging from bactericidal coatings in hospital surfaces to surgical instruments and implants. Among the studied metallic glass forming alloys for antibacterial activity, Zr-Cu-Al-Ag system is of special interest because it contains both Cu and Ag that are well-known antibacterial elements^17^,^18^, and shows excellent glass forming ability which is beneficial for mass production^19^,^20^,^21^. However, antibacterial activity of metals is complex and often involves multiple mechanisms governing the bacterial-material interaction^22^,^23^. Current theories and modeling are unable to predict new alloy compositions that exhibit antibacterial activity. In order to develop metallic glasses with optimized antibacterial properties, which can be paired with other required properties, novel development strategies are required. Recent investigations indicate that combinatorial approaches are promising in accelerating development of new metallic glasses^3^,^24^,^25^,^26^,^27^. Although the combinatorial approach has been used for the development of antibacterial agents in different materials classes^28^,^29^,^30^, its application in metallic alloys remains limited^31^.

Here, we report a combinatorial strategy for the development of Zr-Cu-Al-Ag antibacterial thin film metallic glasses, which enables the simultaneous characterization of a multitude of alloys to identify best-performing compositions. The strategy described here provides a massively parallel approach to reveal the compositional dependence of antibacterial properties, which will help gain new insight into bacterial response to materials and thus, inform the development of new antibacterial materials by design. The developed combinatorial strategy, described herein, is not limited to metallic glasses and can be extended to other material classes.

## Results and Discussion

### Materials library and compositional gradient

**Figure 1:**
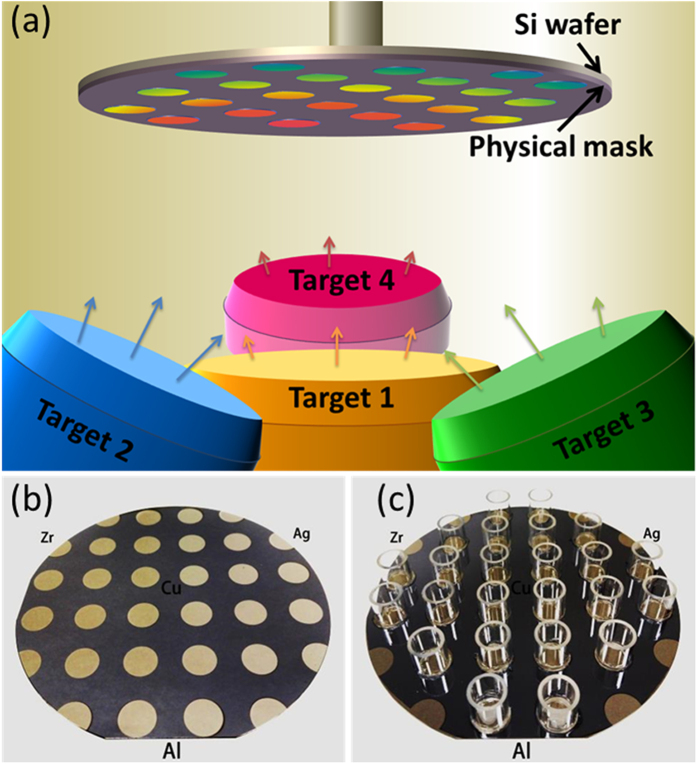
Fabrication of materials library. (**a**) Schematic diagram showing the co-sputtering deposition. Material libraries, Zr-Cu-Al-Ag in the present study, can be prepared by sputtering from four elemental targets. Patched films can be obtained by attaching a physical mask onto Si substrate. Tilting of sputtering guns leads to different target-to-substrate distance which results in decreasing incoming flux across the substrate from near to far away from target, so that a composition gradient can be realized during deposition. (**b**) Appearance of as-deposited materials library. (**c**) Appearance of material library for antibacterial activity measurement. In (**b**,**c**), the relative positions of sputtering targets are indicated.

The combinatorial strategy to screen thin film metallic glasses with antibacterial activity starts with the fabrication of materials library by magnetron co-sputtering deposition, as schematically illustrated in Fig. 1(a), with which the composition gradient of the materials library can be controlled and varied either through tuning the sputter power applied on targets or through the target-to-substrate orientation^3^. Fig. 1(b) shows a typical Zr-Cu-Al-Ag materials library containing 24 alloy patches of ~1 cm in diameter (see also Fig. S1). The thickness of the film ranges from ~500 to ~600 nm, a variation less than 20% (Fig. S2). In order to independently evaluate the antibacterial activity for each composition, the alloys can be further isolated using cloning rings (Fig. 1(c)). In contrast to the conventional rate of alloy generation approximately one alloy per day, the combinatorial synthesis approach enables fabrication of 24 alloys within an hour.

**Figure 2:**
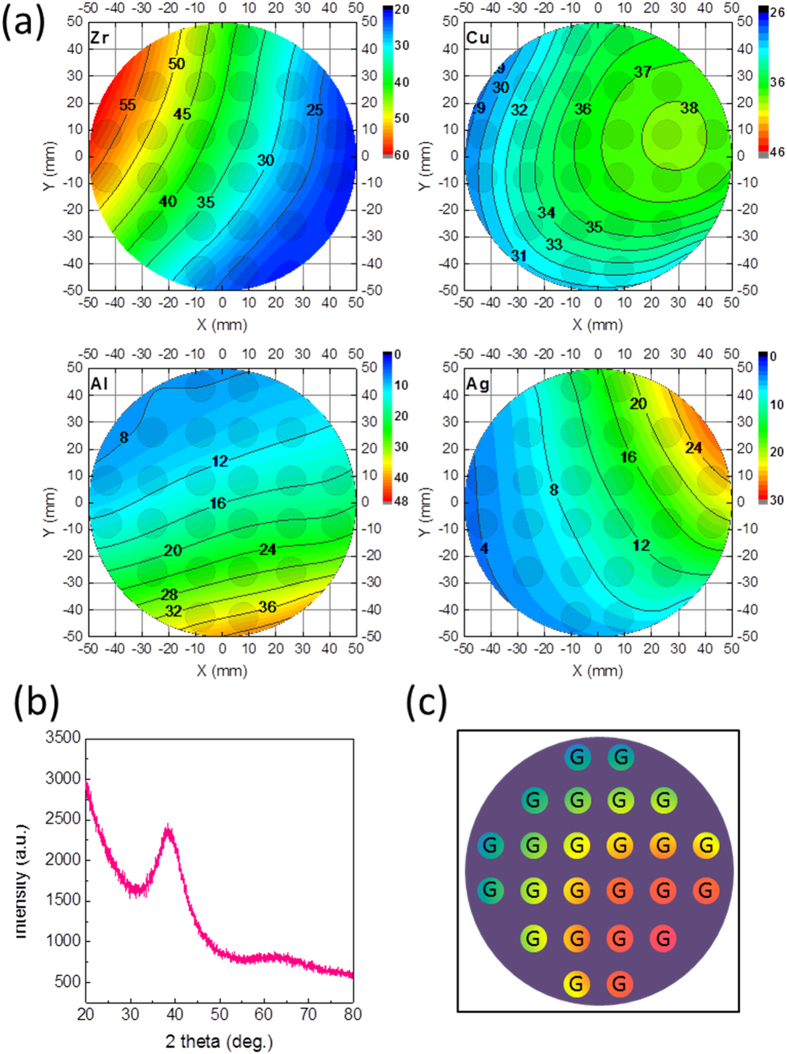
Compositional and structural characterizations of the materials library. (**a**) Contour plots for the concentration of Zr, Cu, Al, Ag, respectively. Relative concentration of each element is indicated by the numbers in the plots, and physical positions of the patched alloys on Si substrate are labeled as circles. (**b**) A representative XRD spectrum for one alloy within the library. The lack of apparent crystalline Bragg peaks indicates that the film alloy is in glassy state. (**c**) Summary of structural characterizations by XRD on all of the alloys within the library. “G” means glass in the summary, indicating that all the alloys are in the glassy state, and the colors of the solid circles are schematic illustration of different compositions.

The composition gradient of the materials library was designed to include the best known glass forming composition range of Zr-Cu-Al-Ag alloy system^20^. Compositional analysis by energy dispersive X-ray spectroscopy (EDX) on each alloy indicated that the materials library spanned a wide range. As shown in Fig. 2(a), variation of the constituent elements can be as wide as 20–55% for Zr, 30–38% for Cu, 8–40% for Al, and 4–25% for Ag. Figure 2(a) shows that there is 3–5% variation in the composition of each patch (~5% for Zr, ~2% for Cu, ~5% for Al, and ~3% for Ag). This in-patch variation arises from the large patch area (~1 cm in diameter) required for the evaluation of antibacterial activity. Despite the variation, compositional dependence of antibacterial activity can be observed when the entire library is considered, as will be shown in the following section. Because the purpose of the current study is to provide proof-of-principle for the ability of the combinatorial system to identify alloys with antibacterial properties, we use the compositions at the center of each patch to represent each alloy.

### Glass formation in the materials library

Zr-Cu-Al-Ag is a well-studied system with excellent glass forming ability^20^. Therefore, a broad composition range is expected to form glass phase under the high cooling rate during sputtering-deposition^32^. We recently reported that automated X-ray diffraction (XRD) is capable of maping out the atomic structure of the materials library^3^,^24^. Figure 2(b) shows the XRD spectrum for one of the alloys in the materials library. As can be seen, the XRD spectrum displays a diffusive pattern without any apparent crystalline Bragg peaks, suggesting the glassy state of the alloy. Although the materials library covers a wide compositional range, our XRD mapping indicates that all the alloys within the materials library are amorphous, as labeled by “G” in Fig. 2(c). Apparently, this glassy range is broader than that by casting^20^, because of the high cooling rate of sputtering with which even marginal glass forming alloys can be fabricated, This suggests that the combinatorial material fabrication approach facilitates the rapid identification of the range of compositions in which glass phases can be obtained. Additionally, a broad glass forming range increases the chances other properties could be optimized without sacrificing glass formation. More importantly, thin film metallic glasses are uniquely suited for antibacterial coatings due to their high wear resistance and durability compared to conventional crystalline metals^10^,^13^.

### Antibacterial activity of thin film metallic glasses

**Figure 3:**
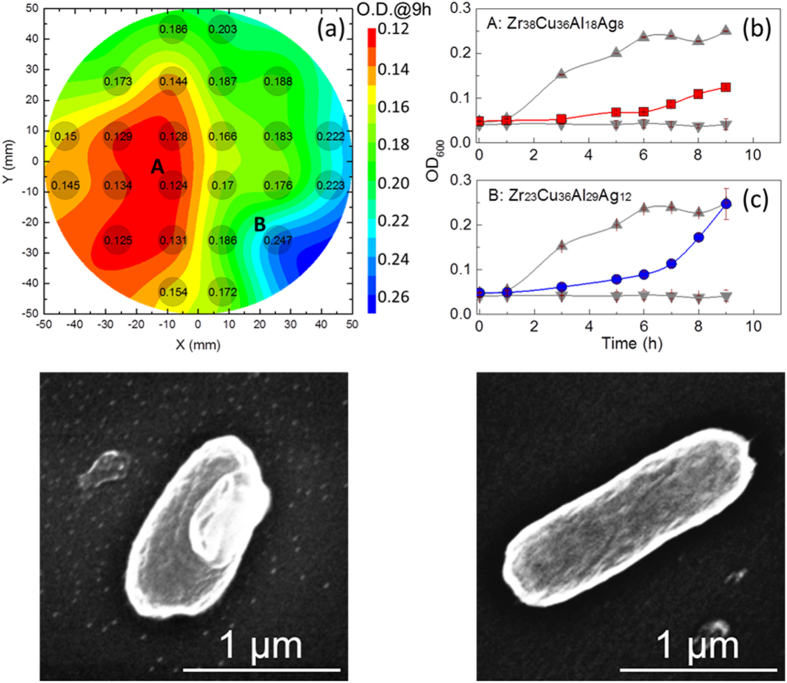
O.D. measurements of the alloys and their effect on bacteria. (**a**) Contour plots based on the O.D. values at 9 hours for all alloys within the library. The O.D. value for each alloy is also included in the plot. (**b**,**c**) are O.D. vs time curves for Zr_38_Cu_36_Al_18_Ag_8_ showing the highest (low O.D. values, as shown by red squares) and Zr_23_Cu_36_Al_29_Ag_12_ showing the lowest (high O.D. values, as shown by blue circles) antibacterial activity. In the O.D. vs time plots, the upwards triangles are for measurements on positive control on which bacteria cells can multiply freely, while the downwards triangles are for measurements on negative controls. (**d**,**e**) are SEM morphologies of E. Coli incubated on alloys of the highest (Zr_38_Cu_36_Al_18_Ag_8_) and lowest (Zr_23_Cu_36_Al_29_Ag_12_) antibacterial activity, respectively.

We evaluated the antibacterial activity of the alloys within our library by culturing E. Coli (SM105) on each alloy composition. Optical density (O.D.) measurements, which serve as a proxy for cell density in the culture medium, were used to study the evolution of the antibacterial activity of thin film metallic glass as a function of time. O.D. measurements are widely used to study the growth of bacteria. An increase in O.D. indicates an increase in the concentration of bacteria in the solution^33^. Here, we employed O.D. measurements to investigate the growth of bacteria incubated on the materials library. Parallel measurements of the O.D. values provide an estimate of the concentration of bacteria, while the shape of the graph indicates the growth trends of bacteria with incubation^33^. O.D. vs. time curves characterizing the multiplication of bacteria are summarized in Fig. S3 for all compositions within the materials library. Figure 3(a) shows the contour plot of O.D. values at 9 hours together with the readings indicated in the plot. This analysis not only reveals the strong yet complex compositional dependence of antibacterial activity, but also facilitates the identification of the best-performing alloys at a glance. For example, the alloy showing the smallest O.D. value can be readily identified as the one of the highest antibacterial activity within the materials library. For all the alloys in the materials library, the O.D. vs time curves appear to be similar at short period of incubation time, e.g. below 6 h (Fig. S3). Only slow increase of bacteria concentration, as reflected by the O.D. values, can be observed. However, longer incubation time reveals the difference in bacteria multiplication behavior. The O.D. vs. time curves for the alloys that showed the highest (Zr_38_Cu_36_Al_18_Ag_8_) and lowest (Zr_23_Cu_36_Al_29_Ag_12_) antibacterial activity are shown in Fig. 3(b,c), respectively. Also included in Fig. 3(b,c) are the O.D. vs. time curves for the measurements on positive control on which bacteria cells can multiply freely, and on negative control which is only culture medium without bacterial. As shown in Fig. 3(b), the increase of bacteria concentration on Zr_38_Cu_36_Al_18_Ag_8_ remains slow after 6 hours (Fig. 3(b)), indicating a prohibited bacteria multiplication even at prolonged incubation time. Nonetheless, bacteria concentration dramatically increases on Zr_23_Cu_36_Al_29_Ag_12_ after long incubation (Fig. 3(c)), suggesting that the alloy is much less efficient in prohibiting bacteria multiplication. The different antibacterial behavior revealed by O.D. measurements is consistent with SEM analyses, as displayed in Fig. 3(d,e). Bacteria on Zr_38_Cu_36_Al_18_Ag_8_ appear to be less elongated, in contrast to that on Zr_23_Cu_36_Al_29_Ag_12_ which retain an elongated rod-like shape of E. Coli. The SEM observations indicate that the alloys within the materials library have different influence on cell behavior of bacteria and thus their multiplication. With the capability of revealing the strong composition dependence of antibacterial activity, our experiments provide a unique combinatorial strategy to identify the best performing alloys.

**Figure 4:**
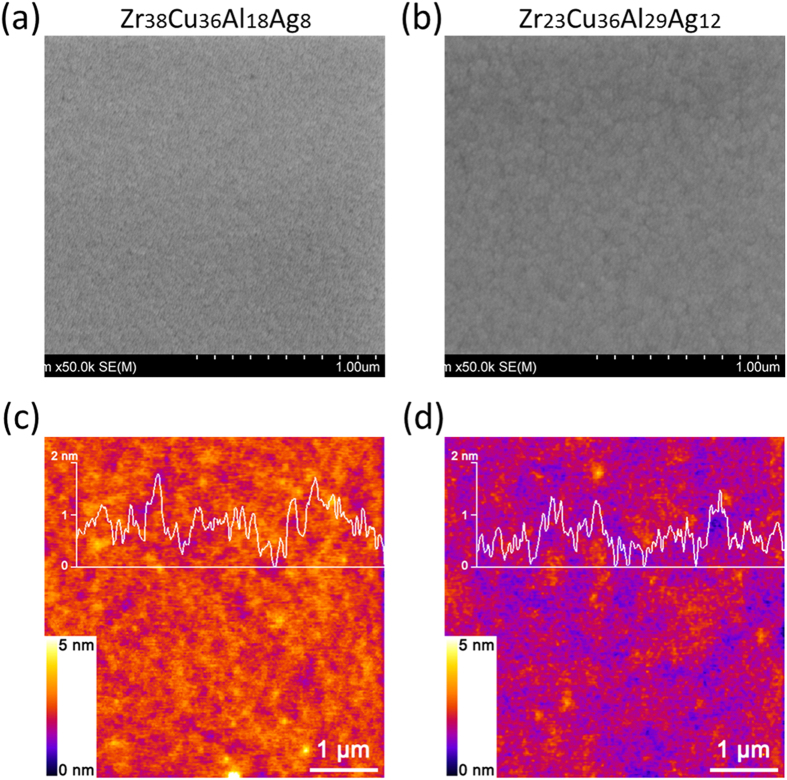
Surface structures observed by SEM and AFM on alloys showing the highest and lowest antibacterial activity. (**a**,**c**) are SEM and AFM images for alloy of highest antibacterial activity, and (**b,d**) are SEM and AFM images for alloy of lowest antibacterial activity. Profiles of the surface roughness are plotted as inserts in (**c**,**d**). The two alloys exhibit similar domain sizes and surface roughness, suggesting that surface morphologies of the films played minor roles in antibacterial activity.

It is known that bacteria-material interactions can be mediated by either surface structures^34^ or metal ion release^23^. To evaluate surface topography dependence of the antibacterial activities observed in the materials library, we characterized the surface structures of the alloys. As shown in Fig. 4(a,b), the alloys exhibit similar SEM morphologies. Furthermore, AFM characterizations indicate that the samples have nearly the same roughness and similar domain sizes, as shown by the surface profiles in Fig. 4(c,d). These results suggest that the variation of antibacterial activities across the materials library is dominated by compositional change, rather than surface structures.

**Figure 5:**
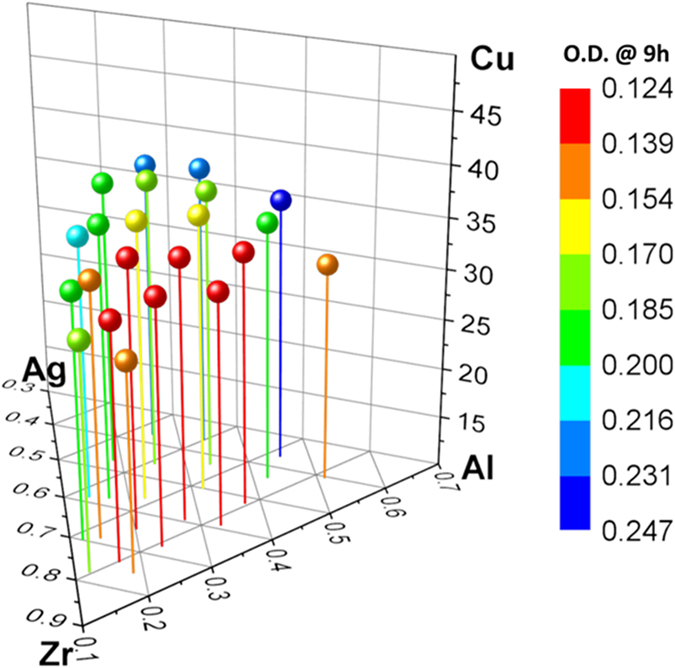
O.D. values at 9 hours as function of relative concentration for the constituent elements, Zr, Cu, Al and Ag, respectively. Since a smaller O.D. value corresponds to higher antibacterial activity, the plots suggests that the better performing compositions are in the range of 30–50% Zr, 33–36% Cu, 10–25% Al, and 5–10% Ag, respectively.

With the combinatorial approach, not only can one quickly screen the best-performing alloy, but also can gain insight into the bacterial-material interaction from an overall perspective. Figure 5 plots O.D. values at the end of 9 hours of incubation against the stoichiometric ratios of each element in the alloy. From the plots, the tendency of how different elements affect antibacterial activity can be seen. Within the materials library, the better performing compositions are in the range of 30–50% Zr, 33–36% Cu, 10–25% Al, and 5–10% Ag, respectively. It can be seen that the antibacterial activity of the Zr-Cu-Al-Ag metallic glasses are sensitive to the content of Cu and Ag, because a small change of their concentration can lead to large variation of antibacterial performance. However, Zr and Al can be varied in a wider range without reducing the antibacterial activity. This observation demonstrates the critical role of Cu and Ag in affecting bacteria behavior and is well consistent with the literature. According to previous investigations^35^,^36^,^37^, antibacterial activity of Zr-Cu-Al-Ag alloys are mainly due to the release of Cu and Ag ions which can damage cell membranes, react with certain proteins, or affect DNA^17^,^18^,^22^,^38^, and thus harming bacteria cell growth. The trends observed in Fig. 5 are possibly associated with the strong composition dependence of ion leaching of metallic glass forming alloys^39^,^40^. While further research into the mechanism of composition-induced changes in bacterial cell density will be helpful, the current combinatorial strategy enables high-throughput screening of thin film metallic glasses for antibacterial activity.

### Conclusion and outlook

In summary, we proposed a combinatorial strategy which enables quick identification of alloy compositions that combine optimized properties. Specifically, we exemplified this strategy here for the combination of glass formation and antibacterial behavior with Zr-Cu-Al-Ag alloy system as an example. Both properties have strong and completely uncorrelated composition dependence, making it very challenging to develop materials that possess both of these optimized properties. We find that in the Zr-Cu-Al-Ag alloy system, the glassy structures can be obtained in a wide composition range, e.g. 20–55% for Zr, 30–38% for Cu, 8–40% for Al, and 4–25% for Ag, by magnetron co-sputtering. With O.D. measurements, we revealed the strong composition dependence of antibacterial activity. Within the materials library, the alloys of higher antibacterial activity are in the range of 30–50% Zr, 33–36% Cu, 10–25% Al, and 5–10% Ag. It was found that antibacterial activity is sensitive to Cu and Ag, while insensitive to Zr and Al.

Antibacterial surfaces have broad applications in healthcare, such as medical tools and disinfecting surfaces. Coating is a widely used strategy to apply antibacterial materials on various structural materials^23^,^41^,^42^. One of the major advantages of metallic glasses is that they exhibit superb properties for both surface (like coatings) and structure^43^. The chemical effects of metallic glasses in killing bacteria, along with the capability of being processed into nanopatterns by either TPF^44^,^45^ or multi-COAD^46^, suggests that surfaces made of metallic glasses are promising candidates for healthcare instruments and surfaces in hospital for disinfecting purpose.

## Methods

The compositional library containing 24 alloys was fabricated by confocal magnetron co-sputtering with DC power supplies using four elemental sputtering targets of purity higher than 99.9% (Zr-281 W, Cu-62 W, Al-183 W, and Ag-20 W). 100-mm-diameter Si wafers (B-doped, p-type, (100) orientation) were used as substrates. A physical metal mask made of 120-μm-thick steel sheet was used to isolate the alloys during deposition. The configuration of targets and substrate is illustrated in Fig. 1(a). The angle of sputtering guns with respect to the substrate normal was kept at 30°. The substrate-to-target distance was 18 cm. The composition gradient of the materials library was controlled by sputtering power applied on the targets on the bases of deposition rate measurement using a quartz crystal thickness monitor. Before deposition, the targets were pre-sputtered to remove possible contamination on target surfaces. The base pressure was lower than 10^−6 ^Pa, and the working pressure was kept constant at 0.3 Pa by flowing high-purity Ar gas. The thickness of the films was controlled by deposition time. The variation in thickness across the library was less than 20%, as shown in Fig. S2. The amorphous nature of the compositional library was characterized by using rapid X-ray diffraction mapping employing a Rigaku SmartLab X-ray diffractometer with a Cu Kα radiation source and Bragg-Berentano geometry. The compositions of the libraries were measured by energy dispersive X-ray spectroscopy (EDX) using an Oxford Instruments X-Max 80 mm^2^ EDX detector in combination with a ZEISS SIGMA field emission scanning electron microscope (SEM). Surface morphology observations were conducted using SEM. Quantitative measurement of surface roughness was carried out using atomic force microscopy (AFM) under ambient conditions. The AFM is a multi-mode nanoscope from Bruker (Veeco), and the measurement was performed under tapping mode with Nanosensors PPP-NCHR-50 tips. The images were recorded with 512 samples per line, and 512 lines were scanned at a scan rate of 0.5 Hz.

The alloys in the materials library were isolated using cloning rings with 1 cm diameter. E. Coli (SM105) in LB broth was incubated with each alloy in the combinatorial library at 37 °C and 180 RPM for 9 hours. For positive control, bacteria with LB broth were incubated on polystyrene plates on which bacteria can multiply freely. For negative control, blank LB broth without any bacteria was incubated in polystyrene plates. O.D. of the bacterial culture was measured at 600 nm using a spectrophotometer at regular intervals. O.D. is a measure of light scattered by living bacteria in culture medium, and increases with increasing bacteria density. An O.D. vs. time curve was plotted for each alloy to analyze the effect of alloy composition on bacterial cell growth. The O.D. readings for all alloy compositions were analyzed. The shape of the graph as well as the final O.D. reading at 9 hours was used to estimate the antibacterial activity of each alloy. To observe the morphology of bacteria, E. Coli in LB Broth incubated on materials library was fixed with 1% glutaraldehyde in 0.1 M Cacodylate buffer for 5 min. The material library with bacteria were then washed with distilled water for 5 min and dehydrated using a series of ethanol washes with increasing concentration from 70% to 100% ethanol. Subsequently, the material library was immersed in hexamethyldisilazane for 15 min. Prior to imaging, the material library was air-dried and then sputter coated with a layer of Iridium. Imaging was completed using an SEM.

## Additional Information

**How to cite this article**: Liu, Y. *et al.* Combinatorial development of antibacterial Zr-Cu-Al-Ag thin film metallic glasses. *Sci. Rep.* **6**, 26950; doi: 10.1038/srep26950 (2016).

## Supplementary Material

Supplementary Information
