# Supplementary material for: Combinatorial development of antibacterial Zr-Cu-Al-Ag thin film metallic glasses
Source: Sci Rep. 2016 May 27;6:26950. doi: 10.1038/srep26950 (PMC4882501; doi:10.1038/srep26950)
Supplement: Supplementary Information [file srep26950-s1.pdf]

Supplementary Information

Combinatorial development of antibacterial Zr-Cu-Al-Ag thin film metallic glasses

Yanhui Liu [1,2,*], Jagannath Padmanabhan [1,3,*], Bettina Cheung [3], Jingbei Liu [1,2], Zheng Chen [1,2], B. Ellen Scanley [4], Donna Wesolowski [5], Mariyah Pressley [1], Christine C. Broadbridge[1,4], Sidney Altman [5], Udo D. Schwarz [1,2,6], Themis R. Kyriakides [1,3,7,#], Jan Schroers [1,2,#]

[1] *Center for Research on Interface Structures and Phenomena, Yale University, New Haven CT 06511, USA*

[2] *Department of Mechanical Engineering and Materials Science, Yale University, New Haven CT 06511, USA*

[3] *Department of Biomedical Engineering, Yale University, New Haven CT 06511, USA*

[4] *Department of Physics, Southern Connecticut State University, New Haven, Connecticut 06515, USA*

[5] *Department of Molecular, Cellular and Developmental Biology, Yale University, New Haven CT 06520, USA*

[6] *Department of Chemical and Environmental Engineering, Yale University, New Haven CT 06520, USA*

[7] *Department of Pathology, Yale University, New Haven CT 06520, USA*

[*] Those authors contributed equally to this work

[#] Corresponding author: Themis R. Kyriakides (themis.kyriakides@yale.edu)

Jan Schroers (jan.schroers@yale.edu)

Supplementary Information

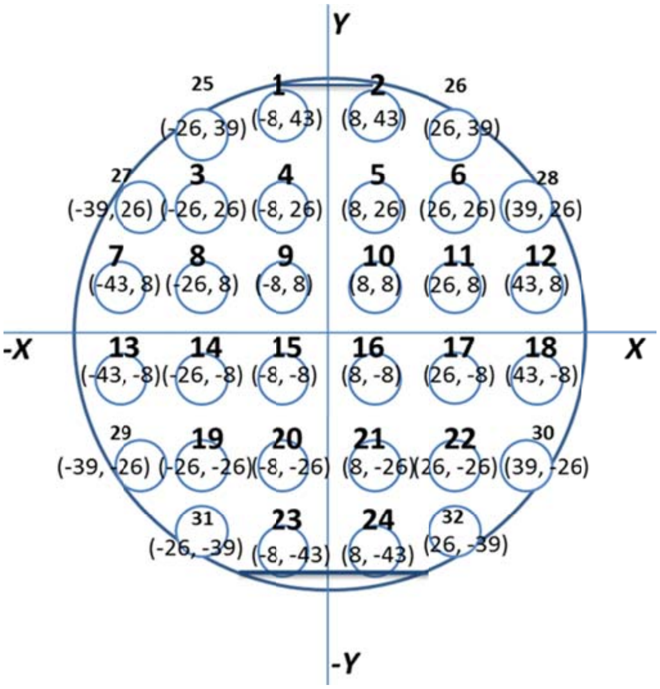

Figure S1 Diagram showing the labels and center coordinates of the patch films on Si substrate. (0, 0) is center of the Si substrate.

Supplementary Information

Table S1 Compositions of the alloys in the material library measured at the center of patches.

Patch#	Zr (%)	Cu (%)	Al (%)	Ag (%)
1	46.1	34.1	7.8	12.0
2	37.6	35.8	8.9	17.7
3	52.5	32.2	8.2	7.1
4	44.6	34.9	9.5	11.0
5	36.4	37.0	10.7	15.9
6	29.2	38.0	11.6	21.2
7	53.5	29.8	12.2	4.5
8	49.1	33.2	11.2	6.6
9	41.0	36.3	12.8	9.9
10	35.0	37.3	14.1	13.7
11	28.2	38.5	15.5	17.9
12	23.2	37.7	16.1	23.0
13	52.5	30.4	12.9	4.3
14	44.4	34.1	15.4	6.0
15	38.3	35.6	17.9	8.2
16	31.5	37.1	20.0	11.5
17	26.7	37.5	21.2	14.6
18	22.3	37.7	21.1	18.8
19	39.6	32.9	22.1	5.4
20	33.8	34.9	24.5	6.9
21	28.0	35.6	27.2	9.2
22	23.4	36.1	29.0	11.5
23	27.6	31.6	35.0	5.8
24	22.4	32.2	38.7	6.7

Supplementary Information

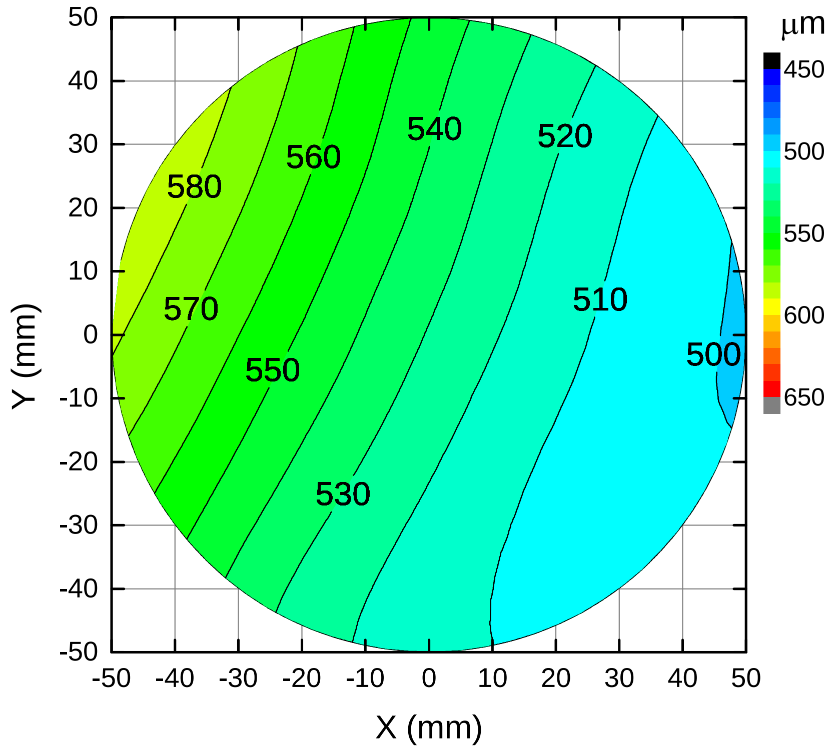

Figure S2 Variation in film thickness across the library.

Supplementary Information

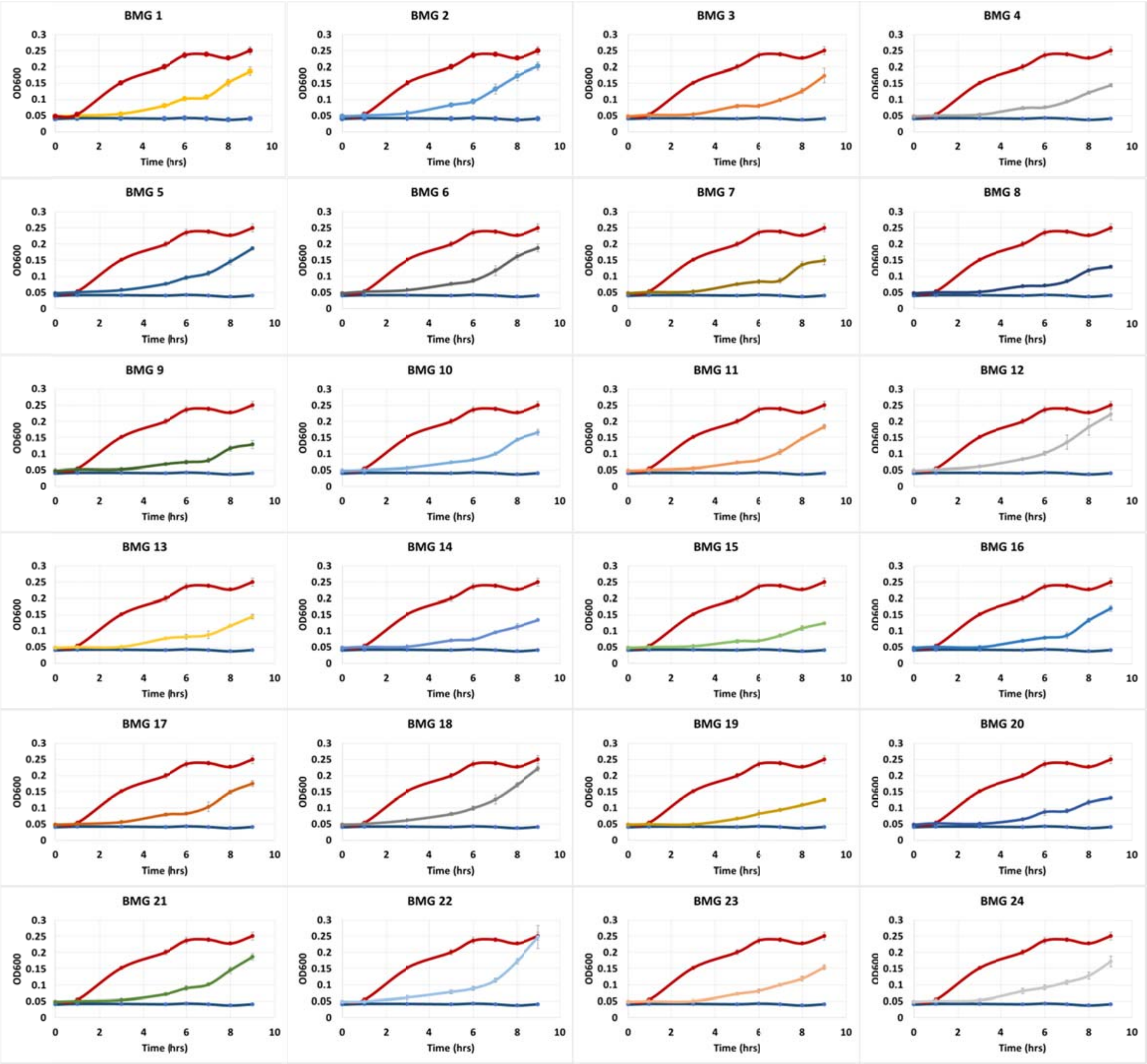

Figure S3 O.D. as function of time for all the 24 alloys in our material library. The numbers correspond to patch labels indicated in Fig. S1.

Supplementary Information
